# Supplementary material for: Genome Sequence Analysis of First Ross River Virus Isolate from Papua New Guinea Indicates Long-Term, Local Evolution
Source: Viruses. 2021 Mar 15;13(3):482. doi: 10.3390/v13030482 (PMC8000771; doi:10.3390/v13030482)
Supplement: Supplementary file 1 [file viruses-13-00482-s001.pdf]

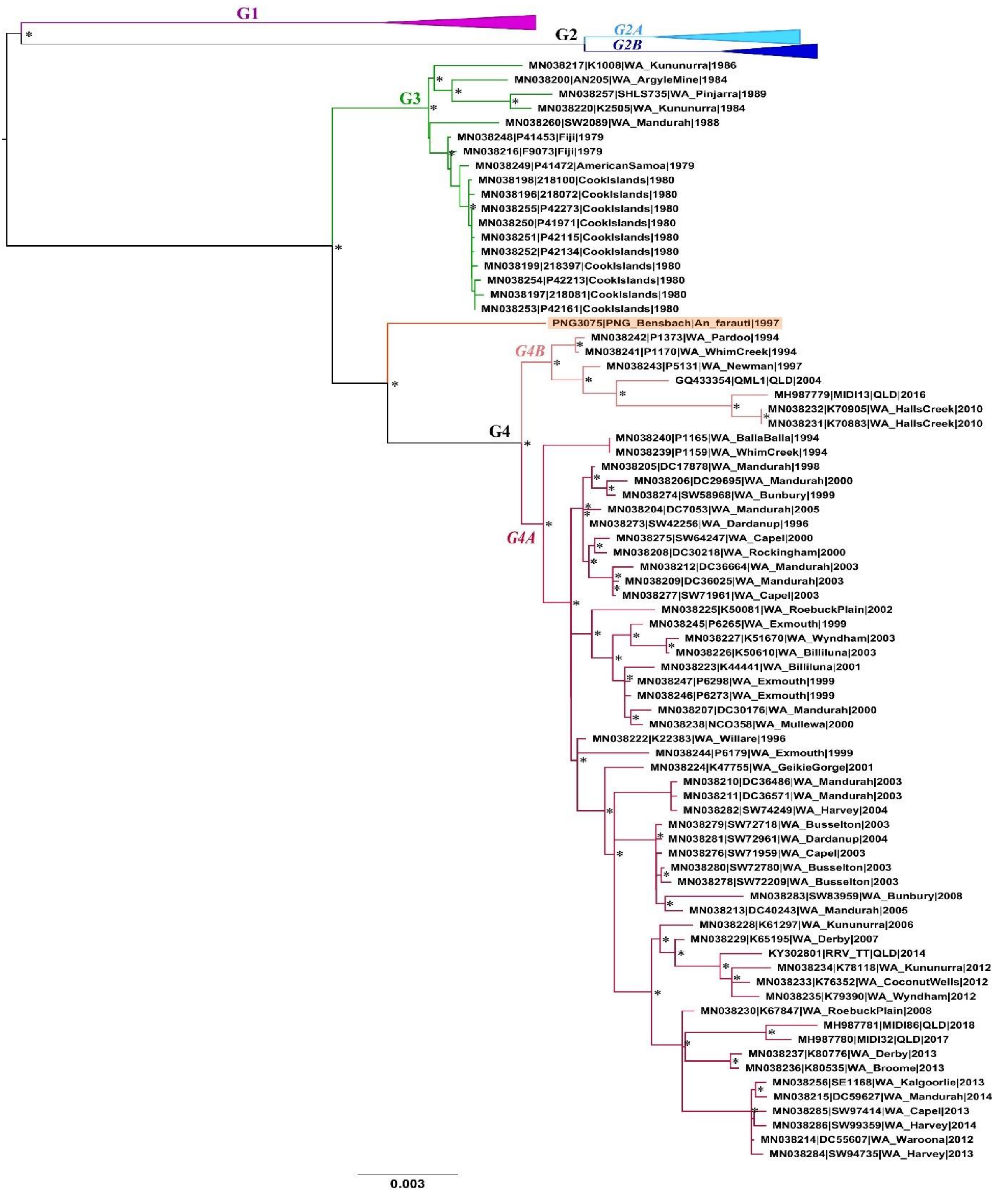

Figure S1: Midpoint rooted maximum likelihood (ML) phylogeny re-constructed from a multiple alignment of a 105-taxon Ross River virus whole genome sequence dataset, including the sequence of the Papua New Guinea isolate, PNG3075 (orange clade). The nomenclature of taxa details the accession number, isolate name, sampling location and sampling year. Bootstrap values over 70% are indicated by asterisks. The Genotype 1 and 2 (G1 and G2) clades have been collapsed as they are consistent with respective clades presented in *Michie et al. 2020* [10].

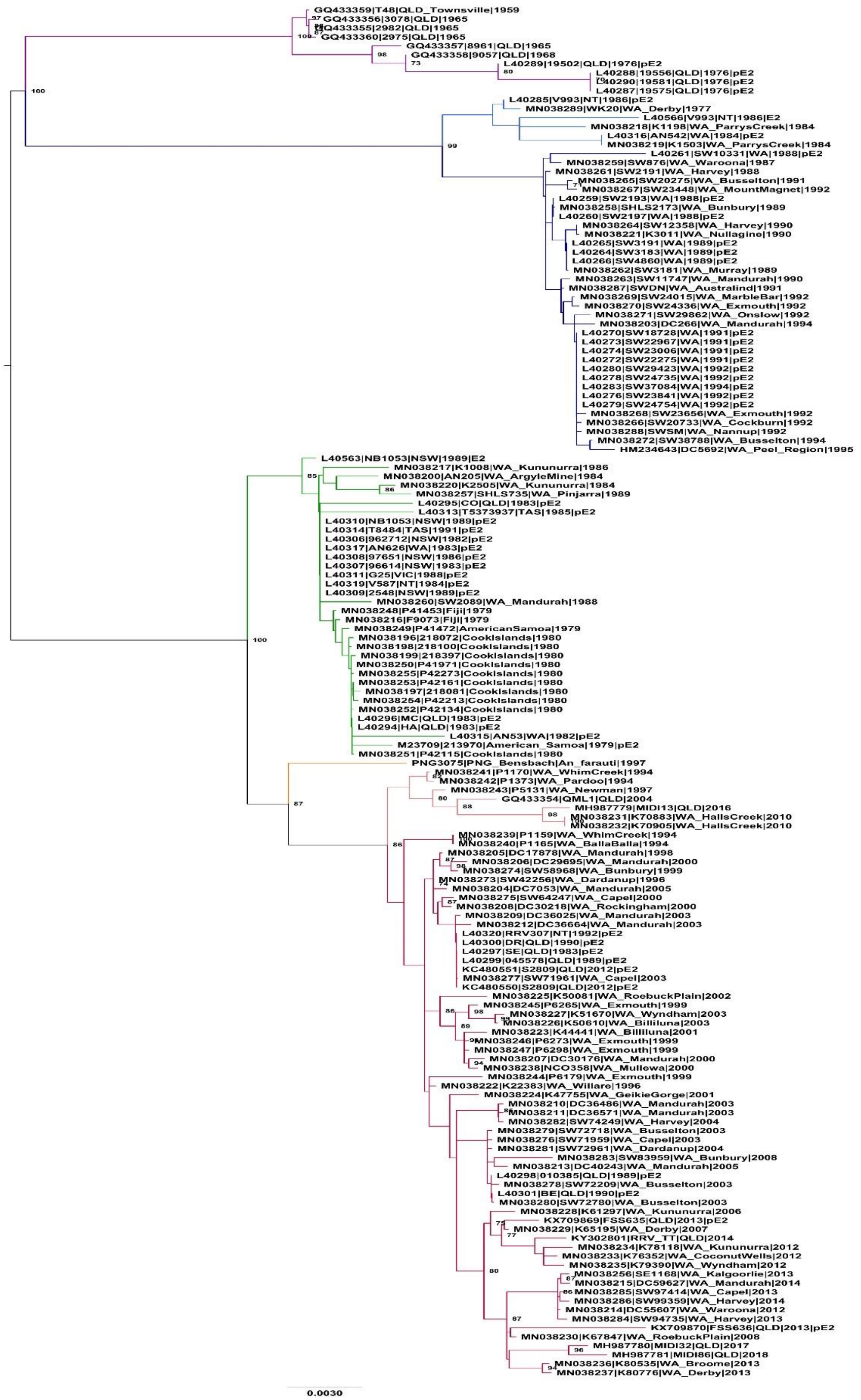

Figure S2: Midpoint rooted maximum likelihood phylogeny of a 105-taxon whole genome Ross River virus dataset aligned with all publicly available whole or partial E2 gene sequences, that are geographically and temporally defined. The PNG clade remains distinct from the G3 (green) and G4 (red) clades in this extended phylogeny. Bootstrap support values over 70% are presented above supported nodes.
